# Non-surgical treatment of hallux valgus: a current practice survey of Australian podiatrists

**DOI:** 10.1186/s13047-016-0146-5

**Published:** 2016-05-04

**Authors:** Sheree E. Hurn, Bill T. Vicenzino, Michelle D. Smith

**Affiliations:** Queensland University of Technology, School of Clinical Sciences, Kelvin Grove, QLD 4059 Australia; Queensland University of Technology, Institute of Health and Biomedical Innovation, Kelvin Grove, QLD 4059 Australia; Division of Physiotherapy, University of Queensland, School of Health and Rehabilitation Sciences, St Lucia, QLD 4072 Australia

## Abstract

**Background:**

Patients with hallux valgus (HV) frequently present to podiatrists for non-surgical management, with a wide range of concerns including pain, footwear difficulty and quality of life impacts. There is little research evidence guiding podiatrists’ clinical decisions surrounding non-surgical management of HV. Thus practitioners rely largely upon clinical experience and expert opinion. This survey was conducted to determine whether a consensus exists among Australian podiatrists regarding non-surgical treatment of HV, and secondly to explore common presenting concerns and physical examination findings associated with HV.

**Methods:**

An online survey was distributed to Australian podiatrists in mid-2013 via the professional association in each state (approximately 1900 members). Podiatrists indicated common treatments recommended, presenting problems and physical examination findings associated with HV in juveniles, adults and older adults. Proportions were calculated to determine the most common responses, and Chi-squared tests were used to examine differences in treatment recommendations according to HV patient age group and podiatrist demographics.

**Results:**

Of 210 survey respondents, 65 % (136) were female and 80 % (168) were private practitioners. Complete survey responses were received from 159 podiatrists for juvenile HV, 146 for adults and 141 for older adults. Seven different non-surgical treatment options were commonly recommended (by >50 % podiatrists), although recommendations differed between adult, older adult and juvenile HV. Common treatments included footwear advice or modification, custom and prefabricated orthotic devices, addition of padding, and muscle strengthening/retraining exercises. Padding was more likely to be utilised in older adults, while exercises were more likely to be prescribed for juveniles. A diverse range of presenting problems and physical examination findings were reported to be associated with HV.

**Conclusions:**

Despite the lack of empirical evidence in this area, there appears to be a consensus among Australian podiatrists regarding non-surgical management of HV, and these recommendations are largely aligned with available clinical consensus documents. Presenting concerns and physical examination findings associated with HV are diverse and have implications for treatment decisions. Management strategies differ across patient age groups, thus any updated clinical guidelines should differentiate between adult and juvenile HV. This study provides useful data to inform clinical practice, education, policy and future research.

**Electronic supplementary material:**

The online version of this article (doi:10.1186/s13047-016-0146-5) contains supplementary material, which is available to authorized users.

## Background

Hallux valgus (HV) is a highly prevalent and progressive musculoskeletal foot deformity, affecting one in three adults over 65 years of age, nearly one in four adults aged 18 to 65 years and 8 % of children (under 18 years old) [1]. Corrective surgery is one form of management, with 6273 orthopaedic procedures performed in 2014 at a direct Medicare Australia cost of $2.5 million [2]. Surgery is often done in conjunction with non-surgical treatments, which may be offered as an alternative to surgery for those with unfavourable medical comorbidities, age [3] or lifestyle factors [4]. Experts agree that non-surgical interventions should be the primary form of treatment offered in juvenile HV, and should always precede operative management, to attempt to reduce symptoms [3–5]. Consequently, patients with HV often present to podiatrists or other health care practitioners for management [6].

In managing HV non-surgically, practitioners and patients are confronted with a wide range of available options [7], and limited research based evidence exists to inform such treatment decisions. The American College of Foot and Ankle Surgeons 2003 [5] consensus statement for initial HV treatment (recommended prior to operative management), includes information on patient education, footwear modifications, orthoses, bunion pads, ice and anti-inflammatory medications. A 2004 systematic review [8] of all interventions for HV (surgical and non-surgical), reported there were only three randomized controlled trials investigating non-surgical interventions for HV (total *n* = 233), concluding at that time there was insufficient evidence supporting the efficacy of treatments studied (foot orthoses or night splints). No recent clinical guidelines or systematic reviews are available surrounding HV treatment, but two small clinical studies have reported equivocal effects of foot orthoses on structural alignment of the hallux (*n* = 54) [9], and for manual therapies versus night splints on pain in HV (*n* = 30) [10]. Compounding the situation of a wide range of available options and limited research based evidence is a lack of understanding of what current podiatric practice offers patients with HV.

Experts advise that treatment should be guided by the patient’s presenting problem [7, 11], however the list of specific concerns reported in those presenting with HV varies widely [12]. Studies have shown that HV may be associated with big toe pain [13], concerns regarding foot appearance, difficulty fitting footwear [14], poor foot function [14, 15] and poor health-related quality of life [16, 17]. It could be assumed that these are the reasons people seek treatment for HV, but research to determine this has not been undertaken [7]. Furthermore, it is unknown whether podiatrists note particular physical examination findings that may also guide their treatment decisions.

An improved understanding of the current practice of podiatrists, along with further information on what brings patients with HV to clinics, will inform planning of clinical trials and practice guidelines. This survey of current practice among Australian podiatrists treating HV was conducted to determine whether there exists a non-surgical treatment consensus, and also whether the current state of practice is aligned with available clinical guidelines. A secondary aim was to explore the most common presenting problems and physical examination findings associated with HV in those seeking treatment from Australian podiatrists.

## Methods

### Study design and participants

This cross-sectional study of Australian podiatrists utilised an online survey distributed between April and August 2013. All members of the Australian Podiatry Association in each state were invited to complete the online survey via email invitation with a link to the SurveyMonkey^©^ platform (approximately 1900 members, based on mailing distribution lists in 2013). To ensure confidentiality of members’ email addresses, administrative staff from each state office distributed the email invitation to members. The survey was further promoted at the Australasian Podiatry Conference (Sydney, June 2013) and remained open for 8 weeks after the conference. Reminder emails were sent on two occasions. Ethical approval was granted by the University of Queensland Medical Research Ethics Committee and QUT University Human Research Ethics Committee (approval numbers 2008001726 and 1300000149).

### Survey instrument

In order to inform the survey design, a focus group was conducted with seven podiatrists, two of whom had specialty training in surgery, and five others who had a special interest in musculoskeletal conditions and/or biomechanics. Levels of experience of focus group participants ranged from 2 to 25 years. All focus group participants provided written informed consent. As an initial prompt question, participants were asked to describe their ‘typical patient’ with HV, and secondly the group was asked to outline a typical treatment plan that might be recommended for HV in four different case scenarios. The focus group was transcribed in full. Lists of presenting concerns and treatment options were extracted from reading the transcript, which were then formatted as fixed response questions (tick boxes) for the online survey. It became clear from reading the transcript that focus group participants would recommend different treatments for children with juvenile HV, adults and older adults. Therefore, fixed response questions were repeated for these different patient types (see Additional file 1).

A preliminary version of the survey was pilot tested on a different group of four podiatrists, who were academics with a broad range of clinical experience. They were asked to provide feedback on the clarity of wording and ease of completing the survey, including time to complete (approximately 15 min). Adjustments were made based on this feedback, prior to distributing the final version of the survey. Changes included minor rewording of questions, as well as adding four opportunities for open responses throughout the survey.

The main body of the final survey instrument (Additional file 1) consisted of sixteen questions (12 fixed response questions and four allowing open-ended responses), divided into four sections: the typical HV patient, the juvenile patient, the adult patient, and the older adult with HV. Within each section survey respondents were asked to select (as ‘tick boxes’) the five most common treatment options, presenting concerns and physical examination findings. Further questions were included in the survey to gather the following participant demographic information: age, sex, location (state/territory) of primary practice, years of clinical experience, practice setting (public/private sector), and full-time or part-time work status. Finally, participants were asked to indicate approximately how many HV cases they had seen in the past month, and whether or not they had completed (or partially completed) specialist surgical training. Two online survey questions (on the first page) indicated participants’ written informed consent prior to completing the survey (see Additional file 1).

### Statistical analysis

Survey data were collected anonymously using the SurveyMonkey^©^ platform. Data were then exported into Microsoft Excel and all fixed response questions were coded dichotomously (yes = 1, no = 0), indicating whether or not the survey participant (podiatrist) chose a particular treatment or identified a particular presenting complaint or physical examination finding for each category of HV patient (typical, juvenile, adult, older adult). Proportions (%) were calculated based on this dichotomous data. Proportions of podiatrists who selected that they would recommend each treatment option were compared in order to reveal the most common treatments. A particular treatment was considered to be ‘common’ if identified as a ‘top 5’ treatment recommendation by >50 % of podiatrists. With regard to the secondary study aim, proportions were similarly compared to reveal the most common concerns from HV patients presenting to podiatrists as well as the most common physical examination findings. Missing data was managed by excluding cases from the analysis. The number of responses per question is reported in the results and tables. Open-ended responses were examined and coded for any common themes that emerged [18]. A descriptive comparison of recommended treatments was undertaken with respect to available clinical guidelines. Chi-squared tests were performed to investigate whether recommendation of any particular treatment (yes/no) differed according to patient type (adult, juvenile, older adult) or was associated with podiatrist demographic variables (age, sex, state, years experience, work setting or part-time/full-time status) or surgical specialty training. This final stage of analysis was performed using the Statistical Package for the Social Sciences (SPSS) Version 22.0 (SPSS Inc., Chicago, IL).

## Results

### Survey responses

In total 210 surveys were returned (11 % response rate based on approximately 1900 members on mailing distribution list). Sixty-nine of the returned surveys were partially completed (33 %); however, no significant demographic differences were identified between completed and partially completed survey responses (Chi^2^*p* >0.05). There were 146 complete survey responses for adult HV, 159 for juvenile HV, and 141 for older adults.

### Participant demographics

Of 210 total survey respondents, 65 % (136) were female and 35 % (74) were male. Table 1 displays participants’ demographic information. All states and territories were represented, albeit with a larger proportion of responses from Queensland (31 %) and Victoria (30 %). A range of podiatrists of varying ages and years of experience were included in the study. Full-time podiatrists represented 70 % of survey respondents, and 80 % worked in the private sector. Eighty-two participants (39 %) reported having seen more than 10 HV cases in the past month, and 25 % reported having seen less than five cases.

**Table 1:** Demographics of 210 survey participants

Participant characteristics	N	%
Sex (Males)	74	35.0
Age categories		
21 to 29	54	25.7
30 to 39	51	24.3
40 to 49	57	27.1
50 to 59	38	18.1
60 or older	10	4.8
State/territory practicing podiatry		
QLD	65	31.0
NSW	28	13.3
VIC	63	30.0
TAS	8	3.8
SA	25	11.9
WA	20	9.5
NT	1	0.5
Years of podiatry experience		
<5	48	22.9
5–10	36	17.1
10–15	34	16.2
15–20	25	11.9
>20	67	31.9
Podiatry practice setting		
Private	168	80.0
Public sector	23	11.0
Other (including >1 location)	19	9.0
Full-time	146	69.5
Part-time	64	30.5
Surgical training (yes)	14	6.7
HV cases seen in the past month		
Less than 5	53	25.2
5 to 10	75	35.7
More than 10	82	39.0

### Treatment of HV

**Fig. 1:**
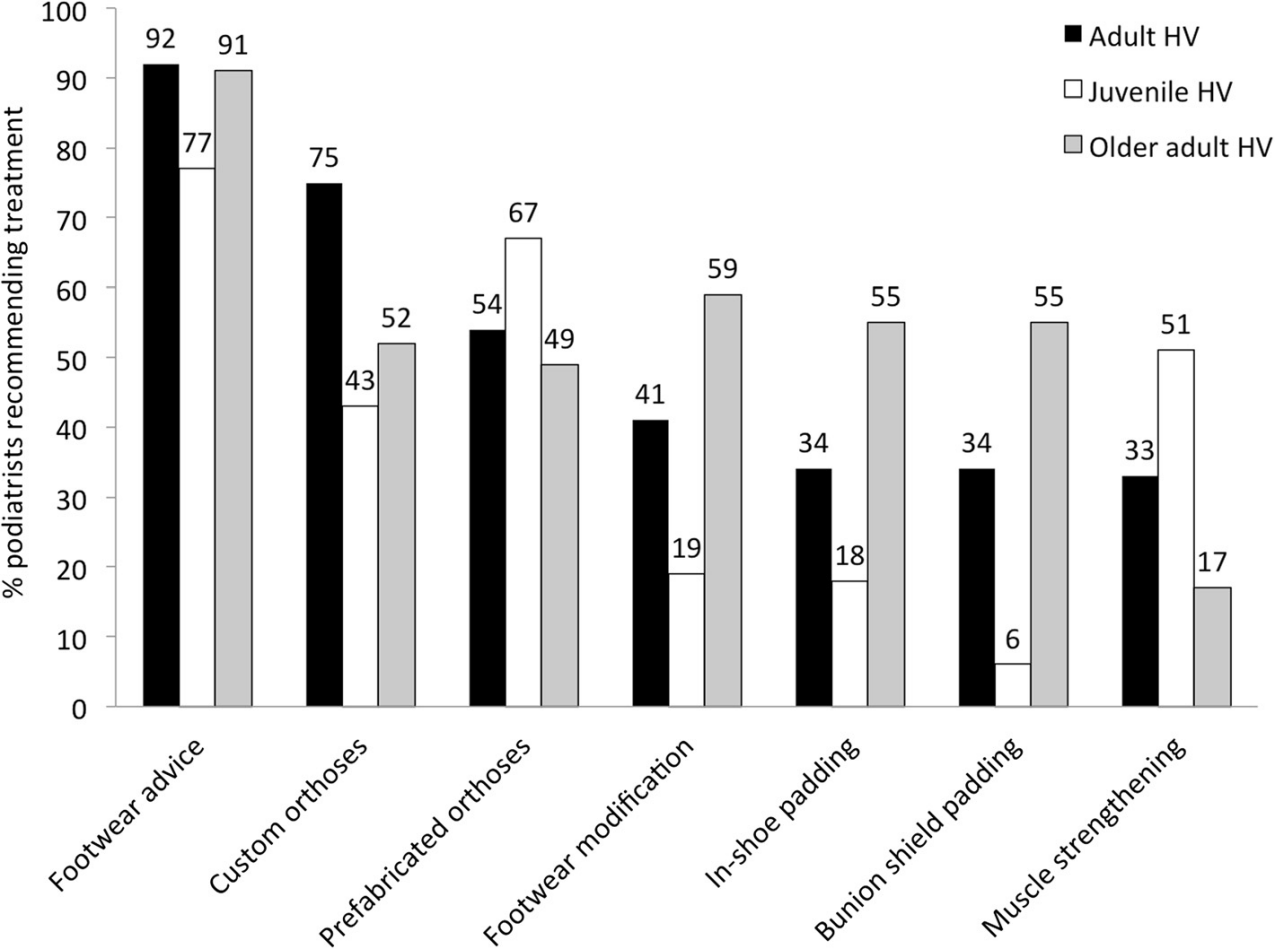
Proportion of survey participants recommending the seven most common treatments across adult, juvenile and older adult HV

Upon preliminary analysis, it became apparent that survey respondents offered very similar responses for the ‘typical’ HV patient compared to an adult patient with HV. Consequently, for clarity, our results are presented for the three categories: adult HV, juvenile HV and older adults. Table 2 shows the proportions of podiatrists who would recommend each different treatment option for HV. Seven treatment options emerged as being commonly recommended by podiatrists for one or more patient types: advice regarding different footwear, custom orthotic devices, prefabricated orthotic devices, footwear modification, in-shoe padding, bunion shield padding, and muscle strengthening/retraining exercises (See Fig. 1).

**Table 2:** Proportions of survey participants recommending different treatment options for HV (values are presented as N (%); bold text highlights >50 % of podiatrists recommending a treatment)

Treatment option	Adult HV (*n* = 146)	Older adult HV (*n* = 141)	Chi^2^ *p*-value^a^	Juvenile HV (*n* = 159)	Chi^2^ *p*-value^a^
Advice regarding different footwear	**134 (92 %)**	**129 (91 %)**	0.69	**133 (77 %)**	<0.001
Orthotic devices - custom	**109 (75 %)**	**74 (52 %)**	<0.001	69 (43 %)	<0.001
Orthotic devices – prefabricated	**79 (54 %)**	69 (49 %)	0.22	**107 (67 %)**	0.001
Modification of existing footwear	60 (41 %)	**83 (59 %)**	<0.001	31 (19 %)	<0.001
In-shoe padding	50 (34 %)	**77 (55 %)**	<0.001	29 (18 %)	<0.001
Bunion shield padding	49 (34 %)	**77 (55 %)**	<0.001	9 (6 %)	<0.001
Muscle strengthening/retraining	48 (33 %)	24 (17 %)	<0.001	**81 (51 %)**	<0.001
Strapping	42 (29 %)	19 (13 %)	<0.001	56 (35 %)	0.07
Muscle stretching	40 (27 %)	23 (16 %)	<0.003	67 (42 %)	<0.001
Lifestyle modification (e.g. exercise/activity)	33 (23 %)	38 (27 %)	0.22	33 (21 %)	0.58
Medication (e.g. NSAIDs)	25 (17 %)	33 (23 %)	0.05	6 (4 %)	<0.001
Night splints	18 (12 %)	11 (8 %)	0.10	60 (38 %)	<0.001
Massage	15 (10 %)	10 (7 %)	0.21	13 (8 %)	0.38
Silicon metatarsal pads	11 (8 %)	28 (20 %)	<0.001	27 (17 %)	<0.001
Dry needling	9 (6 %)	5 (4 %)	0.20	6 (4 %)	0.21

^a^Proportions of participants recommending each treatment were compared across patient age groups, using Chi-squared test and adult HV as the reference group

#### Adult HV

Three treatments clearly emerged as most often recommended for treatment of adult HV: advice regarding different footwear (92 % of podiatrists would recommend), custom orthotic devices (75 %) and prefabricated orthotic devices (54 %).

#### Older adults

In older adult patients with HV, advice regarding different footwear still clearly emerged as a mainstay of treatment (91 % of podiatrists would recommend). Beyond that, there appeared to be less agreement, with a further five treatments emerging as commonly recommended: modification of existing footwear (e.g. stretching) (59 %), in-shoe padding (55 %), bunion shield padding (55 %), custom orthotic devices (52 %) and prefabricated orthotic devices (49 %).

#### Juvenile HV

Slightly different treatment recommendations emerged for juvenile HV patients. Advice regarding different footwear was still the most frequently recommended (77 %), while prefabricated orthotic devices were second (67 %) and muscle strengthening/retraining was third (51 %).

#### Treatment recommendations across patient type

Podiatrists were significantly less likely to offer custom orthoses to juvenile HV patients compared to adult patients (43 % vs 75 %, *p* <0.001), but were more likely to offer prefabricated orthotic devices (67 % vs 54 %, *p* <0.001). Podiatrists were also less likely to utilize such conservative treatments as footwear modification and padding techniques in juvenile HV patients compared to adults. In cases of juvenile HV, muscle strengthening/retraining and stretching exercises (*p* <0.001), as well as night splints (*p* <0.001), were more likely to be recommended.

#### Associations with podiatrist demographics

Compared to those without any surgical specialty training, podiatrists with surgical training were significantly more likely to recommend non-steroidal anti-inflammatory medication for adults (45 % (five out of 11) vs 15 % (20 out of 135), *p* <0.01) and juveniles (25 % (three out of 12) vs 2 % (three out of 147), *p* <0.001). Female podiatrists were more likely to offer bunion shield padding to adults with HV (43 % (40 out of 94) vs 17 % (nine out of 52), *p* = 0.002), and were more likely to recommend strapping for juvenile HV (43 % (43 out of 101) vs 22 % (13 out of 58), *p* = 0.01) compared to male podiatrists. In contrast, male podiatrists were more likely to offer custom orthoses to older adults with HV (67 % (34 out of 51) vs 44 % (40 out of 90), *p* = 0.01). No other podiatrist demographic variables were associated with significant differences in treatment recommendations (age, state/territory, years of experience, public/private sector or part-time/full-time status).

#### Descriptive comparison with clinical guidelines

The top treatments for HV in adults and older adults appear to be aligned with available clinical guidelines published by the American College of Foot and Ankle Surgeons [5]. One notable exception is that very few podiatrists reported that they would often recommend non-steroidal anti-inflammatory medications. The treatments for juvenile HV clearly differed in our survey responses, but to the authors’ knowledge there is no current clinical guideline that differentiates conservative treatments for juvenile versus adult HV.

#### Open-ended survey responses

When open-ended responses were examined, two themes clearly emerged. Thirty-two respondents (15 %) indicated the importance of surgical referral or surgical advice in their treatment pathway. A further ten respondents (5 %) mentioned manual therapy (joint mobilization or manipulation) techniques, which had not been captured in the list of possible responses for treatment options.

### Common presenting concerns in HV

Table 3 displays a list of common presenting concerns associated with HV, and the proportion of podiatrists who identified these to be in their ‘top 5’ observed presenting concerns. The most common presenting concerns associated with HV in adults were trouble fitting shoes (84 % podiatrists reported), big toe pain (82 %), and corns or calluses (75 %). A very similar presentation was reported for older adults with HV: trouble fitting shoes (91 %), corns or calluses (91 %) and big toe pain (76 %). The four most common concerns in juvenile HV emerged as: concern about appearance (89 %), big toe pain (72 %), family history (71 %), and trouble fitting shoes (67 %).

**Table 3:** Proportions of survey participants reporting presenting concerns to be common in HV patients (values are presented as N (%); bold text highlights >50 % of podiatrists reporting)

Presenting complaint	Adult HV (total *n* = 146)	Older adult HV (total *n* = 141)	Juvenile HV (total *n* = 159)
Corns and/or calluses	**110 (75 %)**	**128 (91 %)**	21 (13 %)
Ingrown toenails	39 (27 %)	57 (40 %)	45 (28 %)
Bursitis	35 (24 %)	50 (35 %)	21 (13 %)
Big toe pain	**120 (82 %)**	**107 (76 %)**	**114 (72 %)**
Pain at another site	40 (27 %)	51 (36 %)	63 (40 %)
Trouble fitting shoes	**122 (84 %)**	**129 (91 %)**	**106 (67 %)**
Psychosocial aspects	33 (23 %)	8 (6 %)	73 (46 %)
Concern about appearance	33 (23 %)	58 (41 %)	**142 (89 %)**
Family history	46 (32 %)	32 (23 %)	**113 (71 %)**
Functional limitations	67 (46 %)	**83 (59 %)**	77 (48 %)

### Common physical examination findings in HV

Table 4 presents the list of physical examination findings included in the survey, and the proportion of podiatrists who selected these to be in their ‘top five’ observed findings associated with HV. In adult HV, excessive pronation was commonly noted (50 %) as well as the presence of a bony exostosis (65 %) and degenerative change associated with the first metatarsophalangeal joint (51 %). In older adults, podiatrists more commonly reported the presence of a bony exostosis (70 %) and first metatarsophalangeal joint degenerative change (69 %), in addition to lesser toe deformities (53 %) and corns or callus formation (54 %). Juvenile HV was reported to be associated with excessive pronation (75 %) and a positive family history of HV (62 %).

**Table 4:** Proportions of survey participants reporting physical examination findings to be common in HV patients (values are presented as N (%); bold text highlights >50 % of podiatrists reporting)

Presenting complaint	Adult HV (total *n* = 146)	Older adult HV (total *n* = 141)	Juvenile HV (total *n* = 159)
Bony exostosis	**95 (65 %)**	**99 (70 %)**	46 (29 %)
Pain over medial eminence	49 (34 %)	43 (30 %)	58 (36 %)
Metatarsus adductus	8 (5 %)	7 (5 %)	34 (21 %)
Lesser toe deformity	67 (46 %)	**75 (53 %)**	23 (14 %)
Neuroma	11 (8 %)	2 (1 %)	5 (3 %)
Excessive pronation	**73 (50 %)**	44 (31 %)	**120 (75 %)**
Pes cavus foot type	3 (2 %)	3 (2 %)	9 (6 %)
Anterior ankle impingement	2 (1 %)	0 (0 %)	28 (18 %)
MTP joint pain	69 (47 %)	56 (40 %)	39 (25 %)
Joint subluxation	21 (14 %)	34 (24 %)	19 (12 %)
Concurrent proximal symptoms	9 (6 %)	5 (4 %)	51 (32 %)
Joint hypermobility	10 (7 %)	5 (4 %)	60 (38 %)
1st MTP joint degeneration (OA)	**75 (51 %)**	**97 (69 %)**	7 (4 %)
Corns and calluses	69 (47 %)	**76 (54 %)**	13 (8 %)
Ingrown toenails	8 (5 %)	15 (11 %)	19 (12 %)
Bursitis	17 (12 %)	13 (9 %)	15 (9 %)
Chronic pain	18 (12 %)	18 (13 %)	22 (14 %)
Tight calves	10 (7 %)	3 (2 %)	23 (14 %)
Knee pain	3 (2 %)	1 (1 %)	35 (22 %)
Inappropriate footwear	63 (43 %)	59 (42 %)	25 (16 %)
Poor balance	5 (3 %)	23 (16 %)	15 (9 %)
Muscle weakness	6 (4 %)	8 (6 %)	57 (36 %)
Family history HV	33 (23 %)	17 (12 %)	**98 (62 %)**

## Discussion

The results of this survey have demonstrated that a consensus exists among Australian podiatrists regarding non-surgical management of HV, although typically management recommendations differ between adults, older adults and juveniles with HV. The most common recommendation for all patient types was advice regarding different footwear, recommended by 92 % of podiatrists for adults, 91 % for older adults, and 77 % for juvenile HV. Despite the lack of empirical evidence for the efficacy of orthoses for HV, custom and prefabricated devices are commonly prescribed by podiatrists for HV management. Padding techniques were more likely to be utilised in older adults with HV, while survey respondents were more likely to recommend stretching or strengthening exercises and night splints in juvenile HV.

Current practice of Australian podiatrists managing HV largely aligns with ‘initial treatment options’ outlined by the American College of Foot and Ankle Surgeons in their 2003 clinical consensus document [5], although this organisation acknowledges that their ‘clinical practice guideline’ is no longer up-to-date. Their recommended non-surgical treatment options include footwear advice (wider, lower heeled shoes) or modification, bunion pads, orthoses, ice and non-steroidal anti-inflammatory medications. One notable difference in our study was that use of anti-inflammatory medications was quite low (17 % would recommend for adults, 4 % for juvenile HV and 23 % for older adults), although podiatrists with surgical specialty training were significantly more likely to recommend anti-inflammatory medications for adults and juveniles (*p* <0.01). This could potentially be explained by the fact that at the time of the survey, the vast majority of podiatrists in Australia did not have endorsement to prescribe or supply scheduled medicines. Furthermore, since publication of the American College of Foot and Ankle Surgeons guidelines in 2003, more caution is generally advised regarding the use of non-steroidal anti-inflammatory medications, particularly in older adults [19]. Another difference was in the use of ice for pain relief, as it was not captured by the focus group discussion and therefore was not listed as one of the possible treatment options in the fixed response question format.

This study further investigated whether demographic factors may affect the way podiatrists manage HV. Management recommendations did not differ according to geographical location (state or territory) or years of clinical experience. If these measures are considered to be a proxy for location and year of clinical training, these results suggest that there is a level of consistency in podiatry clinical education across Australia. Interestingly, there were some significant differences between male and female podiatrists in their typical treatment recommendations. Female podiatrists were more likely to offer conservative approaches such as padding and strapping. This may be due to a more sympathetic perspective regarding footwear worn by patients. In contrast, male podiatrists were more likely to offer custom orthoses to older adults.

There was a relatively high level of consensus among podiatrists’ responses for common presenting concerns in HV, but again these differed between adult and juvenile HV. Trouble fitting shoes, pain, and corns or calluses were reported to be most common in adults and older adults, while juveniles appear to present more often with concern about appearance or family history of bunions, in addition to pain and difficulty with footwear. While these presenting concerns clearly stood out as most commonly reported, all concerns listed in our survey were selected by at least 35 % of podiatrists in at least one age group (see Table 3), indicating that the range of presenting problems in patients with HV varies widely.

A more complete picture emerges by examining the physical examination findings reported by podiatrists to be commonly associated with HV (see Table 4). For example, excessive pronation was reported by podiatrists to be commonly found in juvenile HV (75 %) and adult HV (50 %). There is a proposed link between pes planus and HV [20, 21] and so it is unsurprising that orthoses are commonly prescribed for HV, despite limited evidence of their efficacy [9, 22, 23]. Interestingly, different findings appear to be more notable in older adults with HV, with degenerative change or osteoarthritis, lesser toe deformities, and corns or callus becoming more pronounced. It is quite conceivable that as problems secondary to HV develop, such as toe deformities, the treatment plan should be tailored to account for these secondary but associated problems.

This study leads to a number of important outcomes for clinical practice and research. Given the high prevalence and disability associated with HV, up-to-date clinical guidelines are clearly needed for non-surgical management of HV. Such guidelines should differentiate between management of adult HV versus juvenile HV, as the presenting concerns and recommended management of juvenile HV have been shown to differ substantially. It has been recommended previously that a patient’s presenting complaint associated with HV should influence treatment decisions [7]. Based on the widely varying presenting problems associated with HV, as well as different findings on physical examination, caution should be exercised by podiatrists not to apply a ‘blanket approach’ to all patients with this condition. Perhaps treatments to directly relieve symptoms, such as padding and anti-inflammatories, could be more widely utilised by Australian podiatrists to complement other therapeutic strategies such as foot orthoses. While stretching and strengthening exercises appeared to feature in the management of juvenile HV, they were not commonly recommended for adults with HV. Research is needed to determine whether exercise therapy may be beneficial in individuals with HV of all ages. Further research is clearly warranted to form an evidence base for non-surgical management of HV, and the results of this survey could inform the design of such clinical trials, having established what is usual podiatric practice in Australia. Another recommendation for further research is that common problems associated with HV such as pain, callus formation and difficulty with footwear should be considered essential outcome measures in assessing the efficacy of HV management strategies. Previous studies have often used hallux alignment as the primary (and sometimes only) outcome measure [9, 22].

A number of study strengths and limitations should be considered in the interpretation of our results. A strength of this study is that survey responses were returned from a range of podiatrists across Australia, including those of different ages and years of experience. There are a number of limitations that should be considered in making inferences from this data. First, there was a low response rate (11 %), and this could lead to non-response bias. Characteristics of non-responders were not able to be directly investigated; however, podiatrists who completed the survey were compared to podiatry workforce data with regard to geographical location and proportion of males to females. The proportion of females (65 %) was quite similar to podiatry workforce data published by the Podiatry Board of Australia in October 2013 (61 %) [24]. With regard to geographical location, there was a higher response from Queensland (31 %) compared to workforce data (17 %), and a lower response from New South Wales (13 %) compared to workforce data (26 %), while proportions from other states and territories were similar to workforce data [24]. Second, there were a number of incomplete surveys returned, potentially due to the complexity of the survey question format, which required multiple responses for each question. Third, the responses from podiatrists may have been influenced by recall bias, and this study being a cross-sectional survey was not an audit of actual practice, but rather captured podiatrists’ views of their ‘typical’ approach to HV management and ‘typical’ presenting concerns and physical examination findings associated with HV. Even so, the majority of participants reported seeing greater than five cases of HV per month, so this condition was regularly encountered by survey respondents. Fourth, due to the fixed response nature of our question format, our lists of treatments, presenting concerns and physical examination findings were not exhaustive and some treatment options were omitted (e.g. mobilisation/manipulation and ice, as previously discussed). Finally, our survey categorised HV by patient age group, but it should be acknowledged that treatment recommendations may also differ according to whether a patient presents with mild deformity or moderate to severe HV. Similarly, recommendations may differ if a patient has an underlying systemic pathology such as inflammatory arthritis. It may be useful to categorise treatment recommendations in this way; however, this approach was not considered feasible in our survey due to the length and complexity of the survey instrument.

## Conclusion

There appears to be a consensus among Australian podiatrists regarding non-surgical management of HV, with the most common recommendations including footwear advice and foot orthoses, although there is limited evidence for the efficacy of these treatment strategies. Recommendations were largely aligned with existing clinical consensus documents, however management strategies differed according to patient age group. Thus, updated clinical guidelines should differentiate clearly between adult and juvenile HV when addressing non-surgical management. Research is needed to investigate the efficacy of what is current standard practice among Australian podiatrists for non-surgical management of HV (footwear advice and orthoses), in addition to comparative studies investigating the potential efficacy of alternatives treatments such as exercise therapy for HV in different age groups.

## Additional file

Additional file 1: Copy of online survey distributed to study participants. (PDF 1141 kb)
