# Supplementary material for: Non-surgical treatment of hallux valgus: a current practice survey of Australian podiatrists
Source: J Foot Ankle Res. 2016 May 4;9:16. doi: 10.1186/s13047-016-0146-5 (PMC4855349; doi:10.1186/s13047-016-0146-5)
Supplement: Additional file 1: — Copy of online survey distributed to study participants. (PDF 1141 kb) [file 13047_2016_146_MOESM1_ESM.pdf]

PARTICIPANT INFORMATION FOR QUT RESEARCH PROJECT

QUT Ethics Approval Number 1300000149

RESEARCH TEAM

Principal Researcher: Ms Sheree Nix, Lecturer, School of Clinical Sciences, QUT
Associate Researcher: Dr. Michelle Smith, School of Health and Rehabilitation Sciences, University of Queensland and Professor Bill Vicenzino, School of Health and Rehabilitation Sciences, University of Queensland.

DESCRIPTION

This survey is being conducted as part of an ongoing research project investigating hallux valgus, pain and functional outcomes, and conservative treatment.

The purpose of this survey is first to investigate the range of conservative interventions currently utilised by podiatrists in Australia, and second to explore common presenting complaints in patients presenting with Hallux Valgus. This will be achieved through the development and distribution of an online survey.

You are invited to participate in this project because you are a registered podiatrist and member of the Australian Podiatry Association.

PARTICIPATION

Participation will involve completing a questionnaire that will take approximately 10 - 15 minutes of your time. Questions will include your perspectives on HV management and common presentation.

Your participation in this project is entirely voluntary. If you agree to participate you do not have to complete any question(s) you are uncomfortable answering. Your decision to participate or not participate will in no way impact upon your current or future relationship with QUT. If you do agree to participate you can withdraw from the project without comment or penalty. Any identifiable information already obtained from you will be destroyed. However as the questionnaire is anonymous once it has been submitted it will not be possible to withdraw.

EXPECTED BENEFITS

It is expected that this project may benefit podiatrists practising in Australia, by highlighting current practice in treating hallux valgus. Secondarily, this research may also benefit patients presenting with hallux valgus.

RISKS

There are no risks beyond normal day-to-day living associated with your participation in this project.

PRIVACY AND CONFIDENTIALITY

All comments and responses are anonymous and will be treated confidentially unless required by law. The names of individual persons are not required in any of the responses.

Any data collected as part of this project will be stored securely as per QUT’s Management of Research Data policy.

Please note that non-identifiable data collected in this project may be used as comparative data in future projects.

CONSENT TO PARTICIPATE

Submitting the completed online questionnaire is accepted as an indication of your consent to participate in this project.

QUESTIONS / FURTHER INFORMATION ABOUT THE PROJECT

If have any questions or require further information please contact one of the research team members below.

Ms Sheree Nix
Lecturer in Podiatry
School of Clinical Sciences
Queensland University of Technology, Kelvin Grove QLD 4059
t: +61 7 3138 3168
e: s.nix@qut.edu.au

CONCERNS / COMPLAINTS REGARDING THE CONDUCT OF THE PROJECT

QUT is committed to research integrity and the ethical conduct of research projects. However, if you do have any concerns or complaints about the ethical conduct of the project you may contact the QUT Research Ethics Unit on +61 7 3138 5123 or email ethicscontact@qut.edu.au. The QUT Research Ethics Unit is not connected with the research project and can facilitate a resolution to your concern in an impartial manner.

Thank you for helping with this research project.

Please keep this sheet for your information.

*1. Please indicate below that you have read the above information.

- ◯ Yes
- ◯ No

*2. Please indicate your consent to particpate in this research project.

- ◯ Yes
- ◯ No

Demographics

*3. What is your gender?

- Female
- Male

*4. Please indicate your age category.

- 21-29
- 30-39
- 40-49
- 50-59
- 60 or older

*5. In which state do you primarily conduct your podiatry practice?

- QLD
- NSW
- VIC
- TAS
- SA
- WA
- NT

*6. How many years have you practiced podiatry?

- less than 5 years
- 5-10 years
- 10-15 years
- 15-20 years
- 20 years or more

*7. In what setting do you practice podiatry in your primary role/employment?

- [ ] Private Practice
- [ ] Public Sector
- [ ] Research
- [ ] Education

Other (please specify)

*8. In what capacity do you work as a podiatrist?

- Part-time
- Full time

***9. Have you completed (or partially completed) specialist training in podiatric surgery?**

- ◯ Yes
- ◯ No

***10. Approximately how many cases of HV have you seen/treated in the past month?**

- ◯ less than 5
- ◯ 5-10
- ◯ more 10

The following series of questions asks about common presenting complaints, physical examination findings and conservative treatment options offered to hallux valgus patients. You will be asked to answer these questions based on four different scenarios: 1) a typical HV patient, 2) a juvenile HV patient, 3) an adult with HV and 4) an older adult with HV.

The same questions are repeated for each of the four patient scenarios, and your responses to ALL questions will greatly assist this research.

Typical case study of HV presentation and treatment.

The questions below ask you about the presentation of a typical patient suffering from HV, your findings on examination and the most common treatments offered.

*11. Please indicate the five (5) most common complaints of a typical patient presenting with HV (as the primary diagnosis or concern)
Please rate in order from 1 - 5 with 1 being most common and 5 being least common.

	1	2	3	4	5
Corns and/or callouses	◯	◯	◯	◯	◯
Ingrown toenails	◯	◯	◯	◯	◯
Bursitis	◯	◯	◯	◯	◯
Big toe pain	◯	◯	◯	◯	◯
Pain at another site (eg knee, ankle, arches)	◯	◯	◯	◯	◯
Trouble fitting shoes	◯	◯	◯	◯	◯
Psychosocial aspect – work/social expections of footwear	◯	◯	◯	◯	◯
Concern about foot appearance	◯	◯	◯	◯	◯
Family history	◯	◯	◯	◯	◯
Functional limitations	◯	◯	◯	◯	◯

***12. Please indicate the five (5) most common findings on examination of a typical patient presenting with HV.**
Please rate in order from 1 - 5 with 1 being most common and 5 being least common.

	1	2	3	4	5
Bony exostosis	◯	◯	◯	◯	◯
Pain over medial eminence	◯	◯	◯	◯	◯
Metatarsus adductus	◯	◯	◯	◯	◯
Lesser toe deformity	◯	◯	◯	◯	◯
Neuroma	◯	◯	◯	◯	◯
Excessive pronation	◯	◯	◯	◯	◯
Pes cavus foot type	◯	◯	◯	◯	◯
Anterior ankle impingement	◯	◯	◯	◯	◯
MTP joint pain	◯	◯	◯	◯	◯
Joint subluxation	◯	◯	◯	◯	◯
Concurrent proximal problems – scoliosis, leg length difference, postural issues	◯	◯	◯	◯	◯
Joint hypermobility	◯	◯	◯	◯	◯
1st MTP joint degeneration	◯	◯	◯	◯	◯
Corns and callouses	◯	◯	◯	◯	◯
Ingrown toenails	◯	◯	◯	◯	◯
Bursitis	◯	◯	◯	◯	◯
Chronic pain	◯	◯	◯	◯	◯
Tight calves	◯	◯	◯	◯	◯
Knee pain	◯	◯	◯	◯	◯
Inappropriate footwear	◯	◯	◯	◯	◯
Poor balance	◯	◯	◯	◯	◯
Muscle weakness	◯	◯	◯	◯	◯
Family history HV	◯	◯	◯	◯	◯

***13. Please indicate the five (5) most common treatments that you would recommend to a typical patient suffering from HV.**
Please rate in order from 1 - 5 with 1 being most common and 5 being least common.

	1	2	3	4	5
Muscle strengthening/retraining	◯	◯	◯	◯	◯
Strapping	◯	◯	◯	◯	◯
Night splints	◯	◯	◯	◯	◯
Orthotic devices - prefab	◯	◯	◯	◯	◯
Orthotic devices - custom	◯	◯	◯	◯	◯
Silicon metatarsal pads	◯	◯	◯	◯	◯
In-shoe padding	◯	◯	◯	◯	◯
Bunion shield padding	◯	◯	◯	◯	◯
Cortisone injection	◯	◯	◯	◯	◯
Medication (eg NSAIDs)	◯	◯	◯	◯	◯
Massage	◯	◯	◯	◯	◯
Dry needling	◯	◯	◯	◯	◯
Vibration platform	◯	◯	◯	◯	◯
Muscle stretching	◯	◯	◯	◯	◯
Lifestyle modification (eg exercise/activity)	◯	◯	◯	◯	◯
Advice regarding different footwear	◯	◯	◯	◯	◯
Modification of existing footwear (eg stretching)	◯	◯	◯	◯	◯

***14. Is there any other information that you would consider important for a typical HV case ? (not covered in previous questions)**

◯ Yes

◯ No

15. If yes, please comment.

Juvenile (under 18) case study of HV presentation and treatment.

The questions below ask you about the presentation of a juvenile patient suffering from HV, your findings on examination and the most common treatments offered.

***16. Please indicate the five (5) most common complaints of a juvenile patient presenting with HV (as the primary diagnosis or concern)**
Please rate in order from 1 - 5 with 1 being most common and 5 being least common.

	1	2	3	4	5
Corns and/or callouses	○	○	○	○	○
Ingrown toenails	○	○	○	○	○
Bursitis	○	○	○	○	○
Big toe pain	○	○	○	○	○
Pain at another site (eg knee, ankle, arches)	○	○	○	○	○
Trouble fitting shoes	○	○	○	○	○
Psychosocial aspect – work/social expections of footwear	○	○	○	○	○
Concern about foot appearance	○	○	○	○	○
Family history	○	○	○	○	○
Functional limitations	○	○	○	○	○

***17. Please indicate the five (5) most common findings on examination of a juvenile patient presenting with HV.**
Please rate in order from 1 - 5 with 1 being most common and 5 being least common.

	1	2	3	4	5
Bony exostosis	○	○	○	○	○
Pain over medial eminence	○	○	○	○	○
Metatarsus adductus	○	○	○	○	○
Lesser toe deformity	○	○	○	○	○
Neuroma	○	○	○	○	○
Excessive pronation	○	○	○	○	○
Pes cavus foot type	○	○	○	○	○
Anterior ankle impingement	○	○	○	○	○
MTP joint pain	○	○	○	○	○
Joint subluxation	○	○	○	○	○
Concurrent proximal problems – scoliosis, leg length difference, postural issues	○	○	○	○	○
Joint hypermobility	○	○	○	○	○
1st MTP joint degeneration	○	○	○	○	○
Corns and callouses	○	○	○	○	○
Ingrown toenails	○	○	○	○	○
Bursitis	○	○	○	○	○
Chronic pain	○	○	○	○	○
Tight calves	○	○	○	○	○
Knee pain	○	○	○	○	○
Inappropriate footwear	○	○	○	○	○
Poor balance	○	○	○	○	○
Muscle weakness	○	○	○	○	○
Family history HV	○	○	○	○	○

***18. Please indicate the five (5) most common treatments that you would recommend to a juvenile patient suffering from HV.**
Please rate in order from 1 - 5 with 1 being most common and 5 being least common.

	1	2	3	4	5
Muscle strengthening/retraining	○	○	○	○	○
Strapping	○	○	○	○	○
Night splints	○	○	○	○	○
Orthotic devices - prefab	○	○	○	○	○
Orthotic devices - custom	○	○	○	○	○
Silicon metatarsal pads	○	○	○	○	○
In-shoe padding	○	○	○	○	○
Bunion shield padding	○	○	○	○	○
Cortisone injection	○	○	○	○	○
Medication (eg NSAIDs)	○	○	○	○	○
Massage	○	○	○	○	○
Dry needling	○	○	○	○	○
Vibration platform	○	○	○	○	○
Muscle stretching	○	○	○	○	○
Lifestyle modification (eg exercise/activity)	○	○	○	○	○
Advice regarding different footwear	○	○	○	○	○
Modification of existing footwear (eg stretching)	○	○	○	○	○

***19. Is there any other information that you would consider important for a Juvenile HV case ? (not covered in previous questions)**

○ Yes

○ No

20. If yes, please comment.

Adult (18-65) case study of HV presentation and treatment.

The questions below ask you about the presentation of an adult patient suffering from HV, your findings on examination and the most common treatments offered.

*21. Please indicate the five (5) most common complaints of a adult patient presenting with HV (as the primary diagnosis or concern)
Please rate in order from 1 - 5 with 1 being most common and 5 being least common.

	1	2	3	4	5
Corns and/or callouses	◯	◯	◯	◯	◯
Ingrown toenails	◯	◯	◯	◯	◯
Bursitis	◯	◯	◯	◯	◯
Big toe pain	◯	◯	◯	◯	◯
Pain at another site (eg knee, ankle, arches)	◯	◯	◯	◯	◯
Trouble fitting shoes	◯	◯	◯	◯	◯
Psychosocial aspect – work/social expections of footwear	◯	◯	◯	◯	◯
Concern about foot appearance	◯	◯	◯	◯	◯
Family history	◯	◯	◯	◯	◯
Functional limitations	◯	◯	◯	◯	◯

***22. Please indicate the five (5) most common findings on examination of a adult patient presenting with HV.**
Please rate in order from 1 - 5 with 1 being most common and 5 being least common.

	1	2	3	4	5
Bony exostosis	○	○	○	○	○
Pain over medial eminence	○	○	○	○	○
Metatarsus adductus	○	○	○	○	○
Lesser toe deformity	○	○	○	○	○
Neuroma	○	○	○	○	○
Excessive pronation	○	○	○	○	○
Pes cavus foot type	○	○	○	○	○
Anterior ankle impingement	○	○	○	○	○
MTP joint pain	○	○	○	○	○
Joint subluxation	○	○	○	○	○
Concurrent proximal problems – scoliosis, leg length difference, postural issues	○	○	○	○	○
Joint hypermobility	○	○	○	○	○
1st MTP joint degeneration	○	○	○	○	○
Corns and callouses	○	○	○	○	○
Ingrown toenails	○	○	○	○	○
Bursitis	○	○	○	○	○
Chronic pain	○	○	○	○	○
Tight calves	○	○	○	○	○
Knee pain	○	○	○	○	○
Inappropriate footwear	○	○	○	○	○
Poor balance	○	○	○	○	○
Muscle weakness	○	○	○	○	○
Family history HV	○	○	○	○	○

***23. Please indicate the five (5) most common treatments that you would recommend to a adult patient suffering from HV.**
Please rate in order from 1 - 5 with 1 being most common and 5 being least common.

	1	2	3	4	5
Muscle strengthening/retraining	◯	◯	◯	◯	◯
Strapping	◯	◯	◯	◯	◯
Night splints	◯	◯	◯	◯	◯
Orthotic devices - prefab	◯	◯	◯	◯	◯
Orthotic devices - custom	◯	◯	◯	◯	◯
Silicon metatarsal pads	◯	◯	◯	◯	◯
In-shoe padding	◯	◯	◯	◯	◯
Bunion shield padding	◯	◯	◯	◯	◯
Cortisone injection	◯	◯	◯	◯	◯
Medication (eg NSAIDs)	◯	◯	◯	◯	◯
Massage	◯	◯	◯	◯	◯
Dry needling	◯	◯	◯	◯	◯
Vibration platform	◯	◯	◯	◯	◯
Muscle stretching	◯	◯	◯	◯	◯
Lifestyle modification (eg exercise/activity)	◯	◯	◯	◯	◯
Advice regarding different footwear	◯	◯	◯	◯	◯
Modification of existing footwear (eg stretching)	◯	◯	◯	◯	◯

***24. Is there any other information that you would consider important for a Adult HV case ? (not covered in previous questions)**

◯ Yes

◯ No

25. If yes, please comment.

Older Adult (65+) case study of HV presentation and treatment.

The questions below ask you about the presentation of an older adult patient suffering from HV, your findings on examination and the most common treatments offered.

***26. Please indicate the five (5) most common complaints of a older adult patient presenting with HV (as the primary diagnosis or concern)**
Please rate in order from 1 - 5 with 1 being most common and 5 being least common.

	1	2	3	4	5
Corns and/or callouses	◯	◯	◯	◯	◯
Ingrown toenails	◯	◯	◯	◯	◯
Bursitis	◯	◯	◯	◯	◯
Big toe pain	◯	◯	◯	◯	◯
Pain at another site (eg knee, ankle, arches)	◯	◯	◯	◯	◯
Trouble fitting shoes	◯	◯	◯	◯	◯
Psychosocial aspect – work/social expections of footwear	◯	◯	◯	◯	◯
Concern about foot appearance	◯	◯	◯	◯	◯
Family history	◯	◯	◯	◯	◯
Functional limitations	◯	◯	◯	◯	◯

***27. Please indicate the five (5) most common findings on examination of a older adult patient presenting with HV.**
Please rate in order from 1 - 5 with 1 being most common and 5 being least common.

	1	2	3	4	5
Bony exostosis	○	○	○	○	○
Pain over medial eminence	○	○	○	○	○
Metatarsus adductus	○	○	○	○	○
Lesser toe deformity	○	○	○	○	○
Neuroma	○	○	○	○	○
Excessive pronation	○	○	○	○	○
Pes cavus foot type	○	○	○	○	○
Anterior ankle impingement	○	○	○	○	○
MTP joint pain	○	○	○	○	○
Joint subluxation	○	○	○	○	○
Concurrent proximal problems – scoliosis, leg length difference, postural issues	○	○	○	○	○
Joint hypermobility	○	○	○	○	○
1st MTP joint degeneration	○	○	○	○	○
Corns and callouses	○	○	○	○	○
Ingrown toenails	○	○	○	○	○
Bursitis	○	○	○	○	○
Chronic pain	○	○	○	○	○
Tight calves	○	○	○	○	○
Knee pain	○	○	○	○	○
Inappropriate footwear	○	○	○	○	○
Poor balance	○	○	○	○	○
Muscle weakness	○	○	○	○	○
Family history HV	○	○	○	○	○

***28. Please indicate the five (5) most common treatments that you would recommend to a older adult patient suffering from HV.**
Please rate in order from 1 - 5 with 1 being most common and 5 being least common.

	1	2	3	4	5
Muscle strengthening/retraining	◯	◯	◯	◯	◯
Strapping	◯	◯	◯	◯	◯
Night splints	◯	◯	◯	◯	◯
Orthotic devices - prefab	◯	◯	◯	◯	◯
Orthotic devices - custom	◯	◯	◯	◯	◯
Silicon metatarsal pads	◯	◯	◯	◯	◯
In-shoe padding	◯	◯	◯	◯	◯
Bunion shield padding	◯	◯	◯	◯	◯
Cortisone injection	◯	◯	◯	◯	◯
Medication (eg NSAIDs)	◯	◯	◯	◯	◯
Massage	◯	◯	◯	◯	◯
Dry needling	◯	◯	◯	◯	◯
Vibration platform	◯	◯	◯	◯	◯
Muscle stretching	◯	◯	◯	◯	◯
Lifestyle modification (eg exercise/activity)	◯	◯	◯	◯	◯
Advice regarding different footwear	◯	◯	◯	◯	◯
Modification of existing footwear (eg stretching)	◯	◯	◯	◯	◯

***29. Is there any other information that you would consider important for a Older Adult HV case ? (not covered in previous questions)**

◯ Yes

◯ No

30. If yes, please comment.
